# Transdiagnostic symptoms of psychopathology interact with changes in environmental uncertainty

**DOI:** 10.1038/s41598-026-61219-w

**Published:** 2026-08-08

**Authors:** Friederike Elisabeth Hedley, Claudia Lage, Nimrod Hertz-Palmor, Timothy R. Sandhu, Susanne Schweizer, Rebecca P. Lawson

**Affiliations:** 1 Department of Psychology, University of Cambridge, Downing Place, Cambridge, CB2 3EB UK; 2 MRC Cognition and Brain Sciences Unit, 15 Chaucer Road, Cambridge, CB2 7EF UK; 3 School of Psychology, University of New South Wales Sydney, Sydney, NSW 2052 Australia

**Keywords:** Anxiety, Depression, Intolerance of uncertainty, Environmental uncertainty, Network analysis, Cross-lagged panel network, Diseases, Health care, Psychology, Psychology, Risk factors

## Abstract

Uncertainty is a potential driver of poor mental health outcomes, and uncertainty is mounting across domains of daily life. It remains unclear how symptoms of anxiety and depression emerge in response to uncertainty in real-life contexts. The current study investigated how heightened environmental uncertainty interacts with intolerance of uncertainty (IU) to influence mental health symptoms. A total of 301 participants concurrently reported current symptoms of anxiety, depression and IU during higher environmental uncertainty (*t*2) and retrospectively recalled symptoms from a prior period of lower environmental uncertainty (*t*1). Cross-sectional and cross-lagged panel networks were used to identify influential contributors and examine associations across the two reported timepoints. Anxiety, depression and IU symptoms were elevated when environmental uncertainty was higher relative to when it was lower. Three symptom clusters were stable for the two timepoints, but worry became more central to the network at *t*2. IU symptoms exhibited the highest autoregressive effects, indicating strong consistency between levels at *t*1 and *t*2. The item “inhibition of behaviour due to doubt” positively predicted both anxiety and anhedonia. These findings highlight the relevance of worry during periods of increased environmental uncertainty, the predictive value of predispositional IU, and the importance of behavioural inhibition as a potential target to alleviate internalising psychopathology.

**Supplementary Information:**

The online version contains supplementary material available at https://doi.org/10.1038/s41598-026-61219-w.

## Introduction

Uncertainty is increasingly recognised as a key driver of poor mental health outcomes^1^. Cognitive models of internalising psychopathology, such as anxiety and depressive disorders, highlight the role of uncertainty misestimation and intolerance of uncertainty in both the onset and maintenance of these conditions^2,3^. Individual differences in the computational estimation and neural representation of uncertainty have been proposed as risk factors for the development of distinct mental health conditions^4,5^. Yet, to fully understand how different psychopathologies emerge, it is important to integrate information across multiple levels of analysis, spanning genetics, neural circuits, behaviour, self-reports, and environmental context^6^. Here, we focus on a particularly salient but under-examined level: the external environment. We live in a world with pervasive and escalating global uncertainties–including economic instability, political upheaval, armed conflicts, and the climate crisis–that form the backdrop of daily experience for many. Prior research shows that environmental uncertainty, such as that arising in the wake of natural disaster, can negatively impact mental health; however, substantial heterogeneity in how uncertainty and mental health are measured limits comparability across studies^7,8^. Critically, the role of real-world environmental uncertainty in shaping evaluations of one’s retrospective and present self, as well as its contribution to the emergence and maintenance of anxiety and depression in interaction with intolerance of uncertainty, remains poorly understood.

**Fig. 1:**
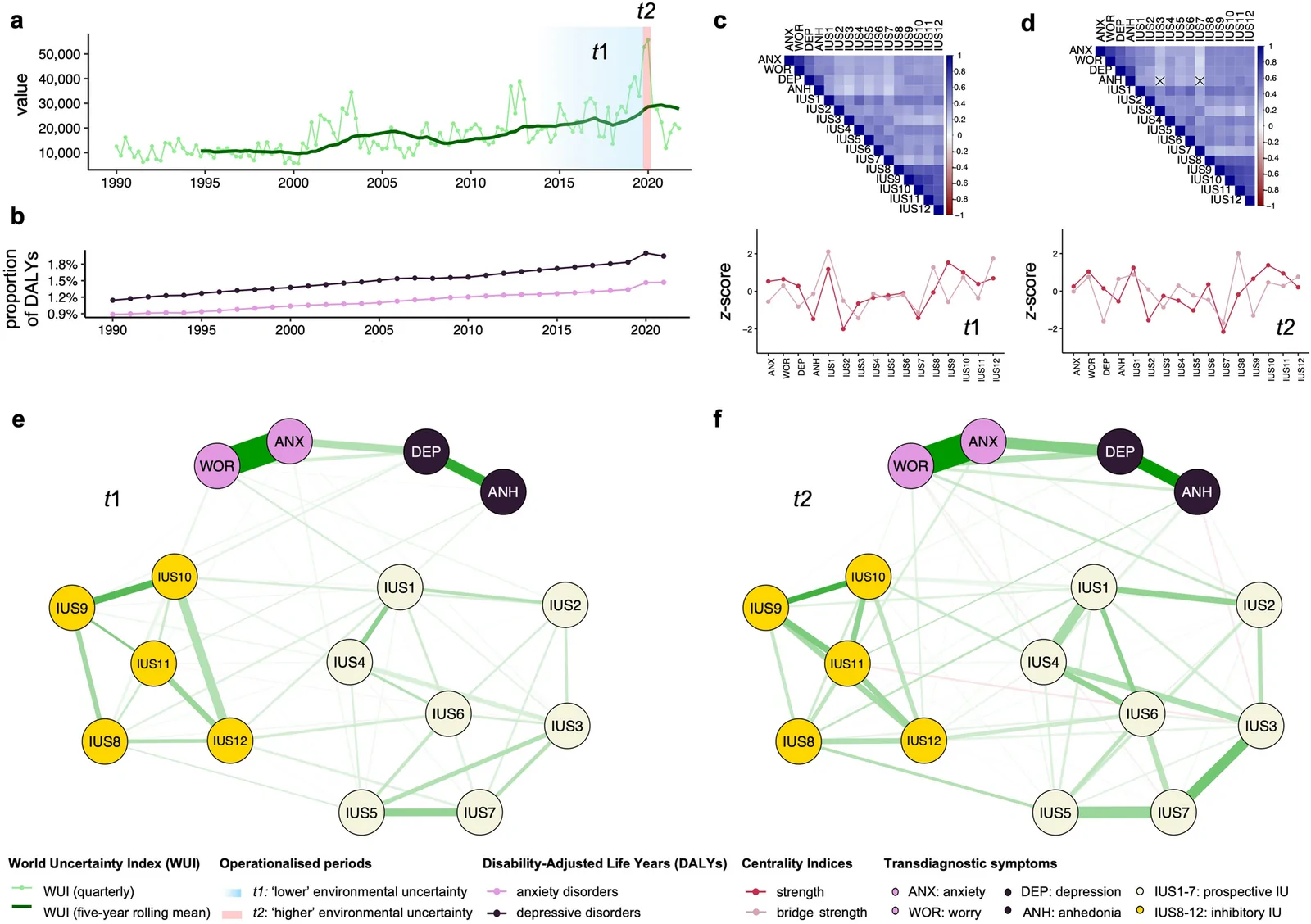
Cross-sectional networks amidst changing environmental uncertainty. (**a**) World Uncertainty Index (WUI), presented in quarterly intervals (light green) and as a five-year rolling mean (dark green), between 1990 and 2021^14^. The index is GDP-weighted for 142 countries. The blue (*t*1) and red (*t*2) shaded sections indicate the periods we operationalise as ‘lower’ and ‘higher’ environmental uncertainty, respectively. Note that we chose these labels to juxtapose the two periods, indicating that they are comparative. Source: World Uncertainty Index. Used with permission. (**b**) Proportion of total disability-adjusted life years (DALYs) for anxiety (pink) and depressive (purple) disorders between 1990 and 2021^15^. Source: Institute for Health Metrics and Evaluation. Used with permission. All rights reserved. (**c**) and (**d**), upper panels, Pearson correlation of the measurements for lower environmental uncertainty (t1) and higher environmental uncertainty (t2). Darker blue reflects greater positive, darker red greater negative correlation. (**c**) and (**d**), lower panels, Centrality indices for *t*1 and *t*2, respectively, with strength (cherry red) and bridge strength (rose) reported in standardised z-scores; larger values reflect greater centrality. (**e**) and (**f**) Cross-sectional networks at *t*1 and *t*2, respectively. Green edges indicate positive and red edges negative relationships. Edge thickness represents the strength with thicker edges indicating stronger relationships. Anxiety-related nodes are coloured in pink (ANX: anxiety/nervousness; WOR: worry), depression-related nodes in purple (DEP: depression/hopelessness; ANH: anhedonia), prospective IU subscale items in beige (IUS1-7), and inhibitory IU subscale items in gold (IUS8-12). IUS: Intolerance of Uncertainty Scale.

One recent example of heightened global uncertainty is the COVID-19 pandemic, referred to as ‘the pandemic’ hereafter, a period marked by a massive surge in the World Uncertainty Index (WUI, Fig. 1a). Fluctuating confinement regulations and evolving epidemiological information contributed to uncertainty across health, occupational, and social domains, arguably affecting mental wellbeing globally. Indeed, this period saw a marked increase in the prevalence of depressive and anxiety symptoms and disorders^9–11^ and their contribution to Disability-Adjusted Life Years (DALYs, Fig. 1b). Though the immediate crisis has passed, the pandemic provides a compelling naturalistic experiment illustrating how large-scale environmental uncertainty can fuel the onset and persistence of psychopathology. From a theoretical perspective, environmental uncertainty can be conceptualised as a chronic increase in ambiguity about future threat and reward contingencies. Within diathesis-stress and cognitive models of anxiety and depression^12,13^, contexts like these are expected to activate cognitive processes, such as worry and rumination, as individuals attempt to restore predictability and control.

Yet not everyone responds to uncertainty in the same way. A key factor shaping individual risk is *intolerance of uncertainty* (IU), traditionally conceptualised as a dispositional tendency to react negatively to uncertain situations across emotional, cognitive, and behavioural domains^3,16^. Higher IU is associated with threat overestimation and an increased likelihood of coping strategies such as rumination, avoidance, or reassurance seeking. Importantly, more recent work has acknowledged IU not only as a dispositional trait for worry and generalised anxiety but also as a transdiagnostic and trans-situational construct that can vary across contexts^17^. Given its links to anxiety disorders, depressive disorders and obsessive-compulsive disorder^18,19^, IU is increasingly recognised as an important treatment target^20–23^. IU can be further subdivided into prospective IU, which reflects the discomfort when anticipating uncertain future events and the desire for predictability, and inhibitory IU, which reflects avoidance behaviour or decision-making paralysis in the face of uncertainty^24^. Together, IU is a dispositional trait, an important risk factor with transdiagnostic and trans-situational features, and a promising treatment target that is central to research on mental disorders, particularly anxiety and depression.

Traditionally, symptom- and disorder-focused approaches have been used to evaluate the structure of mental disorders^25,26^. While these classifications and taxonomies have advanced our understanding of psychopathology, they risk obscuring the complex interplay between symptoms and may fail to capture the dynamic processes that drive comorbidity and clinical outcomes. Network theory offers an alternative perspective on psychopathology, viewing mental disorders as systems of directly interacting symptoms^27^. In this framework, poor mental health may arise within a dynamic system, for example when strong links between symptoms (e.g., depressed mood and worry) form tightly connected nodes within a cross-sectional network. These relationships–or *edges*–are estimated while controlling for all other symptoms, revealing unique associations. Clustering algorithms can identify symptom clusters (e.g., anxious arousal and worry) and cross-cluster connections (e.g., worry and avoidance of surprise), highlighting unique transdiagnostic patterns^28^. A key strength of this approach lies in identifying bridging symptoms and edges that connect distinct clusters, assisting to explain comorbidity. For example, a recent study showed that, during the pandemic, depression-anxiety symptom connections intensified over time among individuals high in IU^29^. However, cross-sectional networks cannot fully capture the *temporal* dynamics of symptom change. Longitudinal research provides important evidence that IU prospectively predicts anxiety- and distress-related outcomes over time, including during and beyond the pandemic, as well as across related domains such as paranoia^30,31^. While these findings underscore the temporal relevance of IU, they typically rely on aggregate symptom measures and therefore do not capture the evolving, symptom-level interactions that may drive psychopathology.

Understanding the extent to which symptom associations change over time is crucial for explaining how environmental uncertainty may destabilise an individual’s sense of self and continuity. Psychological distress can emerge from a mismatch between a retrospective self (a reconstructed, memory-mediated representation of one’s past identity) and a present self (characterised by ongoing experience and perceived diminished agency)^32,33^. When the retrospective self is idealised while the present self is experienced as diminished, this discrepancy may generate distress and contribute to affective dysregulation. Such processes align with cognitive models of internalising psychopathology, in which negatively biased appraisals of the present and future coexist with overly positive reconstructions of the past^34–36^. Examining how symptom associations change across these two self-appraisal contexts requires methods capable of capturing directional evolving dynamics.

Cross-lagged panel network (CLPN) modelling is an emerging method that allows for an analysis of longitudinal effects between symptoms^37^. CLPN models can indicate the direction of potentially causal relationships by examining how nodes (symptom variables) at one timepoint influence symptoms at a subsequent timepoint. In temporal networks, *in-strength* measures a node’s (i.e., symptom) predictability from incoming edges from other nodes at the previous timepoint, while *out-strength* reflects its influence on other nodes via outgoing edges to the next timepoint. Prior work has applied CLPN approaches to anxiety and depression symptom networks, revealing that “nervousness” exhibited highest predictability^38^. However, it remains unclear how global uncertainty interacts with IU to drive mental health symptoms.

In the present study, we investigated how relationships between IU and symptoms of anxiety and depression changed in response to real-world environmental uncertainty. Based on the theoretical frameworks outlined above, environmental uncertainty functions as an external stressor that would interact with dispositional IU to determine the extent to which uncertainty translates into sustained symptom activation across anxiety and depression. Specifically, we first performed cross-sectional network analyses for two timepoints, *lower* and *higher* uncertainty (*t*1 and *t*2, respectively), operationalised as *before* and *during* the pandemic. Importantly, *t*1 measures were obtained via retrospective assessment conducted concurrently with *t*2. Therefore, *t*1 should be interpreted as a reconstructed pre-pandemic self-report rather than as an independently observed baseline. This design introduces potential recall biases, including mood-congruent reconstruction of past symptoms^39^. At the same time, because autobiographical memory is inherently reconstructive, such retrospective appraisals may also be psychologically meaningful. Discrepancies between perceived past and present functioning may contribute to distress, particularly when the present self is experienced as diminished or less agentic. We therefore interpret the retrospective assessment both as a methodological limitation arising from the unprecedented context in which the data was collected, and as a potential window into self-appraisal process under environmental uncertainty.

Our aim was to identify the most influential contributors within networks comprising IU and internalising symptoms, and to compare network structures across time. Second, we used CLPN modelling to examine how symptoms influence one another–and themselves–across the transition from lower to higher environmental uncertainty. Our hypotheses were threefold: Based on prior work highlighting the strong association between worry and IU^40,41^, their longitudinal reciprocal relationship^42^, as well as previous network analyses underlining their relation^43^, we hypothesised worry to emerge as a central node, particularly under conditions of elevated uncertainty (i.e., at *t*2 when environmental uncertainty was higher). Guided by network theory, which conceptualises mental disorders as systems of interacting symptoms^27^, we further hypothesised that heightened environmental uncertainty would lead to higher total network connectivity, reflecting a more densely connected network and measured in a higher value for global strength measure. In addition, drawing on models positioning IU as a transdiagnostic risk factor, we hypothesised that IU^30^, especially its inhibitory aspects reflecting behavioural disruption under uncertainty^44^, would predict increases in internalising symptoms under conditions of heightened environmental uncertainty.

By combining cross-sectional and cross-lagged network analyses, our overall goal was to provide novel insights into the temporal dynamics linking IU to anxiety and mood disorder symptoms, and to identify potential intervention targets for improving mental health outcomes in response to environmental changes.

## Results

### Sample characteristics and descriptive statistics

Summary statistics for demographic information are presented in Table 1. Our final sample was predominantly female (77.74%), with ages ranging from 20 to 81 years (*M* = 41.2, *SD* = 14.3), mostly from the United Kingdom (70.76%). Moreover, 95.34% of participants were in lockdown, and 99% were advised to engage in social distancing at the time of study participation, reflecting the substantial societal changes experienced by participants. Symptom characteristics, as reported via the Patient Health Questionnaire (PHQ^45^) and the Intolerance of Uncertainty Scale (IUS-12^24^), are listed in Table 2. Mean values of anxiety and depression symptoms were greater for higher (*t*2) compared to lower (*t*1) environmental uncertainty (*p* < .001). Furthermore, the proportion of participants who met cut-off values drastically increased from lower to higher environmental uncertainty for anxiety (21.26% → 38.21%, *p* < .001) and for depression (10.96% → 27.24%, *p* < .001). Pearson’s correlations between the symptom variables graphically presented in the upper panels of Fig. 1c, d and numerically in Supplementary Table S1-2.

**Table 1:** Demographic characteristics.

Characteristics		
Age (*M*,* SD*)	41.2	14.3
Gender (*n*,* %*)		
Women	234	77.74
Men	66	21.93
Non-binary	1	0.33
Ethnicity (*n*,* %*)		
English / Welsh / Scottish / Northern Irish / British	213	70.76
Irish	14	4.65
Any other White background	44	14.62
White and Black Caribbean	3	1
White and Asian	7	2.33
Any other Mixed / Multiple ethnic background	2	0.66
Indian	5	1.66
Chinese	6	1.99
Any other Asian background	2	0.66
African	1	0.33
Any other ethnic group	4	1.33
Highest Education (*n*,* %*)		
Primary school	1	0.33
Secondary school/High school	46	15.28
Undergraduate degree	113	37.54
Masters degree	87	28.90
PhD/Doctorate	54	17.94
Context-related measures		
Lockdown initiated	287	95.34
Social distancing recommended	298	99.00

**Table 2:** Symptom characteristics.

Variables	Node	Lower environmental uncertainty (*t*1)		Higher environmental uncertainty (*t*2)		*p*
		M	SD	M	SD	
PHQ-4		2.46	2.90	4.14	3.44	***
Anxiety subscale		1.50	1.85	2.33	1.94	***
Feeling nervous, anxious or on edge	ANX					
Not being able to stop or control worrying	WOR					
Depression subscale		0.96	1.40	1.81	1.81	***
Feeling down, depressed or hopeless	DEP					
Little interest or pleasure in doing things	ANH					
IUS-12		30.72	10.21	32.27	10.82	***
Prospective IU subscale		11.21	5.14	12.07	5.58	***
Unforeseen events upset me greatly	IUS1					
It frustrates me not having all the information I need	IUS2					
One should always look ahead to avoid surprises	IUS3					
A small, unforeseen event can spoil everything, even with the best planning	IUS4					
I always want to know what the future has in store for me	IUS5					
I can’t stand being taken by surprise	IUS6					
I should be able to organise everything in advance	IUS7					
Inhibitory IU subscale		19.52	6.21	20.20	6.38	*
Uncertainty keeps me from living a full life	IUS8					
When it’s time to act, uncertainty paralyses me	IUS9					
When I am uncertain, I can’t function very well	IUS10					
The smallest doubt can stop me from acting	IUS11					
I must get away from all uncertain situations	IUS12					

IUS-12: Intolerance of Uncertainty Scale, responses recorded on a 5-point Likert-scale, with a 7-item subscale for prospective IU, and a 5-item subscale for inhibitory IU.

PHQ-4: 4-item Patient Health Questionnaire, responses recoded on a 4-point Likert-scale, with an anxiety subscale for nervousness/anxiety (ANX) and worry (WOR), and a depression subscale for depression/hopelessness (DEP) and anhedonia (ANH). * *p* < .05; *** *p* < .001.

### Lower environmental uncertainty (*t*1) network

Network analysis of items reported for lower environmental uncertainty (*t*1, see Fig. 1e) identified three clusters (or communities), signalling coherent partitioning of the network into internalising symptom and IU domains. The first community included all four PHQ-items (ANX: anxiety, WOR: worry, DEP: depression, ANH: anhedonia), the second community included the seven items of the prospective IU subscale (IUS1-IUS7), and the third community included the five items of the inhibitory IU subscale (IUS8-IUS12). The modularity of the clustering indicated moderate strength for the reported community structure (*Q* = 0.448). Within this structure, nodes with the higher strength (IUS9, IUS1 and IUS10) were more strongly connected to other nodes in the network, indicating greater involvement in the overall system of associations. Nodes with higher bridge strength (IUS1, IUS12 and IUS8) showed stronger connections across communities, suggesting a potential role in linking IU dimensions with affective symptoms (see Fig. 1c, lower panel, and Supplementary Table S3). The coefficients of stability (*CS*) established via the case-dropping bootstrapping procedure revealed strong and moderate stability for node strength (*CS* = 0.518) and bridge strength (*CS* = 0.385), respectively, supporting the robustness of these estimates. For both networks, accuracy of the edge weights, established via non-parametric bootstrapping, and correlation stability of centrality indices, are graphically shown in Supplementary Fig. S1.

### Higher environmental uncertainty (*t*2) network

For higher environmental uncertainty (*t*2, see Fig. 1f), the three clusters identified were identical to those reported for lower environmental uncertainty (*t*1), indicating a consistent community structure across contexts. The modularity of the clustering indicated a moderately strong community structure (*Q* = 0.415). Nodes with the highest strength were IUS10, IUS1 and WOR, reflecting stronger overall connectivity within the network, while IUS8, IUS1 and IUS12 exhibited highest bridge strength, indicating greater cross-community connections (see Fig. 1d, lower panel, and Supplementary Table S3). Node strength (*CS* = 0.449) and bridge strength (*CS* = 0.415) had moderate stability. Taken together, while cross-sectional network analyses identified consistent symptom clusters across both timepoints, worry specifically showed increased centrality at *t*2. As hypothesised, this suggests a more prominent role of worry within the overall network structure under higher environmental uncertainty.

### Network comparison

Contrary to our hypothesis that heightened environmental uncertainty would be associated with increased overall network connectivity, the Network Comparison Test showed no significant effect for global strength invariance (*t*1 = 7.29, *t*2 = 7.49; *S* = 0.206, *p* = .410). There was also no statistically significant difference in inter-network invariance (*M* = 0.169, *p* = .061), although a trend towards structural variation was observed. Further comparison of the two networks revealed linearity for edge weights and strong correlations for node strength and bridge strength (*rs* > 0.78, *ps* < 0.001, see Table 3).

**Table 3:** Network comparison (*t*1 to *t*2).

Network comparison indices	Test statistic	Adjacency matrices and centrality indices	Test statistic
Network invariance	*M =* 0.169, *p* = 0.061	Edge weight	*r* = 0.781, *p* < 0.001
Global strength invariance Global strength *t*1 = 7.290 Global strength *t*2 = 7.492	*S =* 0.206, *p* = 0.410	Strength Bridge strength	*r* = 0.868, *p* < 0.001 *r* = 0.921, *p* < 0.001

The network comparison test revealed no significant differences between the two networks (*t*1 and *t*2) for inter-network invariance (M) and global strength invariance (S). Strong correlations for edge weight and centralities (node strength and bridge strength) indicate linearity between the networks.

### Cross-lagged panel network (CLPN)

**Fig. 2:**
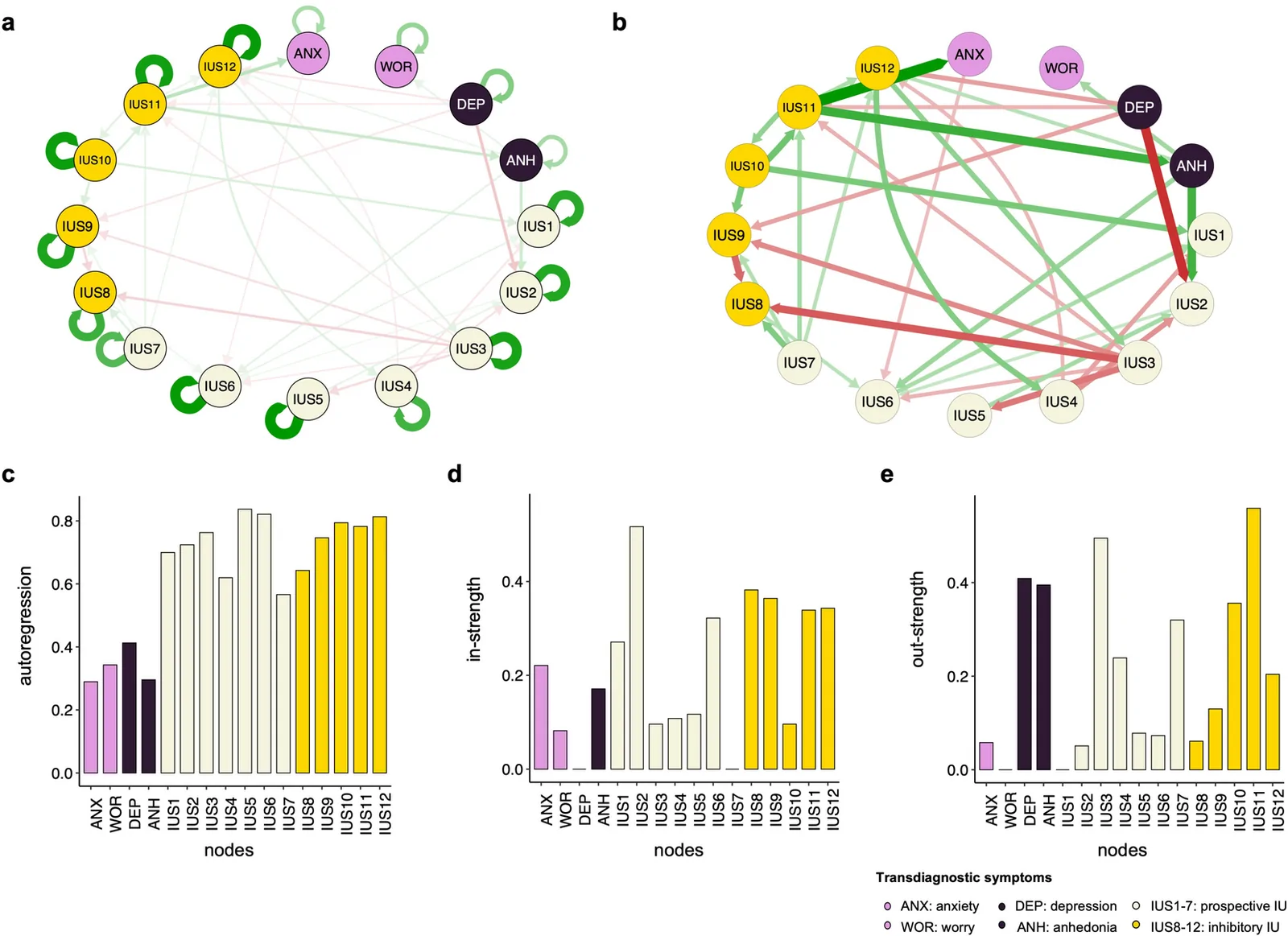
Cross-lagged panel network. (**a**) Full cross-lagged panel network (CLPN) with arrows representing unique evolving dynamics. Green and red edges indicate positive and negative relationships, respectively. Edge thickness represents the strength with thicker edges indicating stronger relationships. Anxiety-related nodes are coloured in pink (ANX: anxiety/ nervousness; WOR: worry), depression-related nodes in purple (DEP: depression/ hopelessness; ANH: anhedonia), prospective IU subscale items in beige (IUS1-7), and inhibitory IU subscale items in gold (IUS8-12). IUS: Intolerance of Uncertainty Scale. (**b**) To ease visual interpretation of the between-node relationships, autoregressive edges were removed from the full CLPN model. (**c**) Autoregression values of the individual measurement items (or nodes). (**d**) Cross-lagged in-strength (i.e., sum of absolute weights of incoming edges) quantified the degree to which each variable (or node) at *t*2 was predicted by other variables (or nodes) at *t*1. (**e**) Cross-lagged out-strength (i.e., sum of absolute weights of outgoing edges) quantified the degree to which each variable (or node) at *t*1 predicted other variables (or nodes) at *t*2. *t*1: lower environmental uncertainty *t*2: higher environmental uncertainty.

Results of the CLPN analysis are shown in Fig. 2a, which includes both autoregressions and standardised between-nodes regressions, visually represented as cross-lagged paths. To ease visual interpretation of the cross-lagged paths, we removed the autoregression and display the network graph in Fig. 2b. The highest positive inter-node regressions were observed from IUS11 to anxiety (*β* = 0.221) and anhedonia to IUS2 (*β* = 0.164), highlighting that the item “The smallest doubt can stop me from acting*”* emerged as the strongest predictor of anxiety symptoms, supporting the hypothesised predictive role of inhibitory IU. The highest negative values were found for depression to IUS2 (*β* = − 0.179) and IUS3 to IUS8 (*β* = − 0.142). The highest autoregression (see Fig. 2c) values were observed for two prospective IU items (IUS5: *β* = 0.837; IUS6: *β* = 0.821) and two inhibitory IU items (IUS12: *β* = 0.813; IUS10: *β* = 0.794), while the lowest autoregression values were assigned to the four PHQ items (ANX: *β* = 0.289; ANH: *β* = 0.296; WOR: *β* = 0.343; DEP: *β* = 0.413). In other words, all autoregressive effects in IU outperformed those of anxiety and depression. All values of the standardised regression matrix for the CLPN model are shown in Supplementary Table S4. Analysis of centralities showed that IUS items had the highest cross-lagged in-strength, indicating they were the most predictable (IUS2 = 0.517; IUS8 = 0.382; IUS9 = 0.364; IUS12 = 0.343; see Fig. 2d). Among the items with the highest cross-lagged out-strength–meaning those that most impact other nodes–were two IUS items and both depression items (IUS11 = 0.559; IUS3 = 0.495; DEP = 0.409; ANH = 0.395, see Fig. 2e). Interestingly, while only negative inter-node regressions were reported for depression (DEP), only positive inter-node regressions were found for anhedonia (ANH). The complete set of in-strength and out-strength values are reported in Supplementary Table S5.

Together, CLPN results highlighted that *behavioural inhibition due to doubt* (IUS11) positively predicted both anxiety and anhedonia. IUS items had the highest predictability and the highest autoregressive effects, indicating they were more stable than mental distress across varying levels of environmental uncertainty.

## Discussion

The current study established networks for intolerance of uncertainty (IU), anxiety and depression symptoms before and during a surge in real-world environmental uncertainty. Overall, our results show that in a period of heightened uncertainty, when mental distress was elevated, worry became more central in the network, and IU items served as strong autoregressive predictors. Specifically, the symptom *inhibition of behaviour due to doubt* positively predicted anxiety and depression symptoms. This examination is of great relevance given the alarming prevalence rates of anxiety and depressive disorders amidst an increase of all-encompassing uncertainties worldwide. Furthermore, results of this study speak to the importance of how IU, as a proposed transdiagnostic factor, relates to internalising psychopathology symptoms in the face of uncertain environments.

In the lower environmental uncertainty context, the nodes with the highest strength and bridge strength belonged to the IU construct, while amidst higher environmental uncertainty, *worry *had one of the highest node strengths. Whereas times with comparably lower environmental uncertainty showed lower internalising symptom centrality, the higher IU centrality indicates an increased vulnerability in people already concerned about potentially unexpected major events. This aligns with the notion of IU being a shared factor of emotional disorders^46^, meaning it exists, beyond a mere correlate, as a predisposition to negatively perceive and respond to uncertainty^47,48^. Among the anxiety and depression symptoms, worry emerged to play a more central role in times of heightened global uncertainty. As more central symptoms are thought to be crucial for disorder maintenance^49^, this finding further aligns with increased occurrence of depression and generalised anxiety symptoms during the outbreak of the global pandemic^50,51^. Furthermore, establishing key symptoms and connecting symptoms in depressive-anxiety symptom network models has important clinical implications, as targeting these may enhance treatment effectiveness in patients and preventative care in high-risk individuals. Our findings highlight that during times of higher global uncertainty, the *cognitive* component of anxiety, that is anxious apprehension, operationalised as worry, becomes more relevant. Worry presents itself as a future-orientated, often uncontrollable, cognitive feature, with hyper-fixation on potential threat^52,53^. We show that worry not only increases but also becomes more influential in the transdiagnostic symptom network during higher levels of environmental uncertainty. Its heightened centrality suggests that worry develops increased influence on other nodes (e.g., depression or IU symptoms), potentially amplifying its effects within the overall network.

Direct comparison of the cross-sectional networks revealed high correlations between network edges and centrality indices. This implies that the network structures stayed intact when comparing the lower and higher uncertainty contexts, despite increased overall symptom severity. Consistent with this pattern, network invariance testing did not indicate significant differences between the two networks, suggesting some degree of stability in symptom relationships across time. However, because the lower-uncertainty assessment was retrospective, the observed similarity may also reflect shared reconstruction processes or mood-congruent recall bias. Although there was a general trend towards greater overall connectivity in the higher environmental uncertainty network, this difference did not reach statistical significance, potentially due to limited statistical power. Future research using prospective longitudinal designs is needed to clarify whether symptom network structure changes across periods of differing environmental uncertainty. Larger samples are also required to determine whether discrepancies between retrospective and present self-representations meaningfully influence observed network differences.

Using a community detection algorithm, we identified three communities for both cross-sectional networks, which corresponded with the entire set of anxiety and depression symptoms, as well as the two domains of the Intolerance of Uncertainty Scale (IUS; inhibitory IU and prospective IU subscales). This not only validates the factor structure of the IUS but also highlights the close interconnection and comorbidity of anxiety and depression symptomatology^24,45,54^. In other words, the algorithm did not establish separate communities for anxiety and depression symptoms. In the context of recent developments towards empirically-based research frameworks and classification of mental disorders (e.g., Research Domain Criteria^6^, and Hierarchical Taxonomy of Psychopathology^55^) as well as computational modelling approaches^56–58^, our findings reinforce the value of transdiagnostic approaches that focus on symptom-level interactions rather than traditional diagnostic boundaries. The overlapping community structure observed in our networks reflects the empirical reality of shared mechanisms and symptom co-occurrence across disorders, supporting efforts to refine classification systems and tailor interventions to underlying processes rather than diagnostic labels. However, future work should test the robustness of this community structure using more granular and comprehensive measures of anxiety and depression symptoms.

Results from the cross-lagged panel network show that autoregressions of the IU items were higher than anxiety and depression symptoms. Autoregressive paths highlight the stability of individual items between the timepoints of measure, reflecting idiosyncratic impacts across *t*1 and *t*2. In other words, IU variables outperformed internalising psychopathology variables in their value in predicting anxiety and depression symptoms. This finding supports previous work that highlighted how IU is an important risk factor for internalising psychopathology during heightened global uncertainty^29^. IU has been conceptualised as a trait-like, trans-situational variable^17,59^. Thus, this personality-type feature may hold important, yet overlooked, predictive value beyond the traditional psychopathological symptoms (e.g., anxiety and depression) in informing the onset and maintenance of mental health difficulties. Our results show that this holds especially true in the face of increased environmental uncertainty. Indeed, in the context of the all-encompassing uncertain environments many individuals now face, our findings underscore the importance of targeting IU in both preventative and therapeutic interventions, and of using it as a marker of vulnerability before major stressors occur^3,60^. This perspective aligns with recent theoretical work emphasising the need for treatment specificity when addressing situational uncertainty-related distress, including interventions that extend beyond the individual level to incorporate systemic (policy-level) changes^22^. Moreover, consistent with research identifying IU as a candidate mechanism for the development, persistence, and treatment of mood and anxiety disorders, our findings further support positioning IU at the centre of transdiagnostic models of internalising psychopathology.

Furthermore, notable relationships between individual symptoms emerged in the modelling investigating evolving dynamics. A key aspect of interpreting network paths in the CLPN is that they reflect conditional predictive relationships–that is, the influence of each variable at *t*1 on *t*2, while controlling for all other variables at *t*1. One particularly salient finding was that *inhibition of behaviour due to doubt* (IUS11) at times of lower uncertainty (*t*1), positively predicted anxiety and anhedonia during higher uncertainty (*t*2). This aligns with previous research implicating behavioural inhibition as a risk factor for anxiety^61^ and with neurocognitive models emphasising behavioural and cognitive avoidance as central mechanisms of anxiety under uncertainty^2^. Our findings suggest that behavioural inhibition rooted in doubt may represent a treatment target in both cognitive behavioural therapy and preventative mental health strategies.

Within cognitive therapy approaches for anxiety disorders, there is emphasis on attention bias modification^62^. These methods support goal-engagement factors in the context of attention but could be extended to disengaging individuals from harmful doubts. Similarly, by promoting acceptance, mindfulness, values, and committed action, therapy approaches such as Acceptance and Commitment Therapy (ACT^63^) help individuals build psychological flexibility. This flexibility is crucial for managing self-doubt and taking steps towards a more fulfilling life, making ACT a particularly promising treatment approach for uncertain situations. Beyond individual-level interventions, these findings also point to the relevance of upstream structural factors that shape exposure to uncertainty, such as socioeconomic instability, information environments, and broader societal stressors. Future work should therefore integrate psychological and public health perspectives to inform multi-level preventive strategies targeting both individual vulnerability and environmental sources of uncertainty that may be amenable to structural change.

Interestingly, we also found that anhedonia positively predicted *frustration of not having relevant information* (IUS2), whereas depression predicted the same item negatively. Individuals who reported high levels of anhedonia were more frustrated in situations involving a lack of relevant information; at the same time, individuals who reported high levels of depression were less frustrated in these contexts. This distinction aligns with theoretical models that differentiate motivational difficulties (such as reduced approach behaviour and reward sensitivity in anhedonia) from the broader cognitive and affective profile of depression, which may include hopelessness, resignation, and reduced cognitive engagement with the external world^64,65^. Individuals high in anhedonia may still exhibit a desire for goal-directed information, despite blunted hedonic responsiveness—and may feel more acutely frustrated when such information is unavailable. Conversely, individuals with more pervasive depressive symptoms may be less responsive to uncertainty due to learned helplessness. This dissociation suggests that frustration in uncertain contexts may reflect a residual or thwarted form of goal pursuit, preserved in some internalising profiles but diminished in others.

The findings of this network analysis should be interpreted in light of several limitations. First, the sample was a predominantly female community (opportunity) sample, which supports generalisability but limits applicability to clinical population. Although a substantial proportion of participants reached cut-off values for both depression and anxiety symptoms during higher environmental uncertainty, all measures were based on self-report rather than diagnostic assessments. In the context of ongoing debates about optimal approaches to psychopathology assessment^6,25,55^, future studies should incorporate categorical, dimensional, and transdiagnostic methods and examine these networks in clinical samples, as well as more gender-balanced and culturally diverse populations, given known population differences (e.g., prevalence of anxiety and depression symptoms are higher in women^66,67^ and such differences may influence both the structure of symptom networks and the temporal dynamics observed). Second, both timepoints were assessed concurrently, introducing the possibility of recall bias^68,69^ for the retrospective timepoint (*t*1), particularly given the emotional salience and duration of the pandemic. Although a design incorporating prospective pre-pandemic baseline data would have been preferable, and comparisons between the *t*1 and *t*2 network structures should therefore be interpreted appropriate caution, perceived discrepancies between past and current functioning may themselves exacerbate distress. This may be especially true when individuals perceive their current self as less capable or agentic than their past self, providing insight into self-appraisal processes under changing environmental uncertainty. Moreover, because both timepoints were reported simultaneously, cross-lagged paths reflect associations structured by instructional framing rather than true prospective effects and should therefore not be interpreted causally. Relatedly, the operationalisation of pre- versus during-pandemic periods as lower versus higher uncertainty was based on macro-level indices (i.e., WUI) and does not capture individual differences in perceived uncertainty, which may be more proximally related to psychological responses. Nevertheless, almost all participants in the present study were in lockdown and were advised to engage in social distancing at the time of study participation, reflecting the substantial social and personal changes they were experiencing. Third, we did not assess broader dispositional traits (e.g., neuroticism) or prior life experience (e.g., trauma, adversity)^70,71^ limiting our ability to evaluate the specificity of IU and its developmental or sociocultural context. Additionally, the use of the brief PHQ-4, likely constrained symptom granularity, and the clustering of anxiety and depression items may reflect measurement limitations rather than true structural overlap. Future work should employ more comprehensive and item-rich measures of internalising psychopathology to allow for finer-grained network analyses and more robust community detection. Finally, there are ongoing methodological challenges in psychological network modelling^72,73^, including questions regarding appropriate time lags^37,74^. Indeed, relationships between symptoms may occur at shorter or longer time periods, as parameter estimates vary as a function of the time interval between measurements^75^. To address this, future research could employ high-frequency sampling techniques, such as daily diary methods or ecological momentary assessment, to track real-time fluctuations in IU and emotional responses to environmental stressors. Furthermore, the two-wave CLPN implemented here does not decompose observed variance into stable between-person and time-varying within-person components. As a result, the estimated autoregressive and cross-lagged effects may conflate stable individual differences with true within-person change over time. Approaches such as the panel graphical vector-autoregression model^76^ explicitly separate within- and between-person variance, but require a minimum of three measurement occasions to reliably estimate temporal dynamics. Because the present study included only two (retrospectively assessed) timepoints, implementation of such models was not feasible. Future work should employ more extensive longitudinal designs with several measurement occasions to fully separate these effects.

Employing network analyses on transdiagnostic symptomatology, we investigated how real-world environmental uncertainty interacts with intolerance of uncertainty (IU) to drive mental health symptoms. Cross-sectional networks highlighted that worry became more central in times of higher environmental uncertainty, while cross-lagged panel network modelling revealed that inhibition of behaviour due to doubt, reported during times of relative global stability, positively predicted both anxiety and anhedonia. Notably, autoregressive effects suggest that during periods of greater certainty, internalising symptoms are poorer predictors of future distress. In contrast, transdiagnostic traits like IU are more stable and, crucially, serve as predictors of symptoms and mental distress, even when distress itself is not necessarily present. This suggests that trait-level cognitive vulnerabilities, such as IU, can anticipate the onset or worsening of symptoms—even when overt distress is not yet observable. In an era defined by global and personal uncertainty, these findings offer new insights into who is vulnerable, when, and why. By identifying IU-related processes as both persistent and predictive, our results underscore the value of targeting these mechanisms in early intervention and treatment.

## Methods

### Participants

Ethical approval for the study was granted by the Cambridge Psychology Research Ethics Committee (PREC), ethics number: PRE.2019.110. The experiment and all methods were carried out following the declaration of Helsinki. All participants provided informed consent prior to data collection through an online form. As there is no straight forwarded approach for power calculations in psychological networks^77^, we based our sample size on previous network modelling work using IU measures, targeting a minimum of 250 participants^43,78^. After removal of incomplete datasets (*N* = 123), our final sample included 301 participants. Participants completed a survey administered on the Research Electronic Data Capture (REDCap^79^), and anonymised data was collected between April and May 2020.

### Measures

Participants completed several questionnaires and for each we asked them to indicate their answers for two timepoints. For anxiety and depression measures, we captured their responses for “the last two weeks” (i.e., during the pandemic) and “the two weeks before the Covid-19 outbreak”. For personality trait measures, to anchor participants’ pre-pandemic responses in a common and interpretable way, they were instructed to “indicate how much these statements were characteristic of you before the Covid-19 outbreak”. Thus, *t*1 was assessed retrospectively and collected concurrently with *t*2. Although retrospective self-report is commonly used in psychological research^80,81^, it is vulnerable to recall bias and mood-related memory confounds^82^. Accordingly, we refer to *t*1 as retrospectively reported *lower* environmental uncertainty and *t*2 as current *higher* environmental uncertainty and interpret comparisons between these assessments with appropriate caution.

### Patient health questionnaire

We first report data of the 4-item Patient Health Questionnaire (PHQ-4^45^), for which responses are recorded on a Likert-scale, ranging from 0 = *not at all* to 3 = *nearly every day*. The PHQ-4 includes two subscales: an anxiety subscale measuring self-reported anxiety/nervousness and worry, and a depression subscale measuring depression/hopelessness and anhedonia. Cut-off scores of ≥ 3 are suggestive of clinical anxiety and depression, respectively.

### Intolerance of uncertainty scale

We also collected self-reported data from the short-version of the Intolerance of Uncertainty Scale (IUS-12^24^), for which responses are recorded on a Likert-scale, ranging from 1 = *not at all characteristic of me* to 5 = *entirely characteristic of me*. The IUS-12 includes two subscales: a 7-item subscale measuring prospective IU, which reflects the desire for predictability and reduced uncertainty (e.g., “Unforeseen events upset me greatly”), as well as a 5-item subscale measuring inhibitory IU, which captures one’s propensity to display avoidance behaviour when faced with uncertainty (e.g., “When it’s time to act, uncertainty paralyses me”).

### Data analysis

All data analyses were conducted in RStudio (R version 4.4.3).

### Cross-sectional networks

To examine the structure of the relationship among variables, partial correlation network analysis was performed. Here, each node represents a variable (i.e., individual symptom/IU trait) and edges capture the relationships between nodes. In this context, a regularisation technique was used to penalise model complexity as to remove edges that are likely to be spurious, after controlling for all other variables^77,83^. Hence, edge weights represent the partial correlation coefficients between nodes, and variables independent from one another would have an edge weight of zero with no connection being drawn between these nodes. We first established regularised partial correlation networks based on standardised *z*-scores of the variables for both timepoints (i.e., lower and higher environmental uncertainty) via the *bootnet* package^77,84^. Specifically, we estimated our networks through a least absolute shrinkage and selection operator (LASSO^85^), using the extended Bayesian information criterion (EBIC^83^) to estimate a sparse graphical model with a tuning hyperparameter (*γ*) of 0.5. This is a relatively stringent value of this parameter that minimises type-I errors and, at the expense of sensitivity, strengthens confidence in the emerging edges. This approach is recommended for polychoric correlation of polytomous data sets, like those obtained via questionnaires using Likert-scales in this study. We visualised the networks via the *qgraph* package^86^.

### Community detection

To establish communities (or clusters) of highly connected nodes, we used the Walktrap algorithm of the *igraph* package^87^. Here, communities are detected by performing random walks on a network and the partition with maximized modularity, as a measure of clustering strength, is subsequently selected. We then examined the modularity index *Q*, which provides a quantification of the intracommunity connectivity^88^.

### Centrality, accuracy and stability

We next assessed the centrality indices of node strength (i.e., sum of the absolute weights of every edge) and node bridge strength (i.e., sum of absolute weights of edges across clusters) using the *qgraph* and *networktools* packages^86,89^. Importantly, centrality indices provide information about the relative importance of nodes within the network structure. In particular, node strength reflects how strongly a given variable is directly connected to all other variables in the network, with higher values indicating nodes that are more strongly associated with the broader symptom system. Bridge strength indicates the extent to which a node connects different symptom communities/clusters and therefore reflects its potential role in linking distinct domains (e.g., anxiety- and depression-related clusters). We did not report closeness and betweenness which have been shown to exhibit spurious associations among symptoms^90^. For calibrated interpretations of network model results, it is important to examine accuracy measures by assessing fluctuations observed from sampling variability in edge weights. For each network model, we thus established the accuracy of the edge weights via non-parametric bootstraps with 2,000 samples each. The non-parametric bootstrapped resampling approach estimates confidence intervals (CIs) between the bootstrapped and sample means, with smaller and larger CIs suggestive of heightened and poorer precision of the edges, respectively. If a CI includes the value zero, this indicates that different node strengths (or edge weights) do not significantly differ from one another. We also assessed the stability of centrality estimates via case-dropping bootstraps with 2,000 samples each. The quantification of this stability was established using the correlation coefficients of stability (*CS*) which indicates whether centrality indices (i.e., strength and bridge strength) maintained stability when proportions of cases were progressively dropped (e.g., 10%, 20%, 30%). *CS* values stand for the maximum proportion of dropped samples, given that the correlation between original centrality indices could reach 0.7 at a 95% probability. A *CS* > 0.25 implies moderate stability and a *CS* > 0.5 implies strong stability^77^.

### Network comparison

Similarity of the two networks (lower and higher environmental uncertainty) was explored through correlations of the adjacency matrices and centrality indices (a value of 1 indicates perfect linear relationship, i.e., the same structure; a value of 0 indicates no relationship). Next, we used the NCT function of the *NetworkComparisonTest* package^91^ with 1,000 permutations and Bonferroni-Holm correction^92^. Network structure invariance, global strength (summed value of all network edges) and differences in edge weights were analysed.

### Cross-lagged panel network (CLPN)

Finally, we conducted a two-step CLPN analysis of the data collected for lower (*t*1) and higher (*t*2) environmental uncertainty^37^. Central to CLPN modelling is the feature of directed paths across timepoints (in our example *t*1 and *t*2), reflecting connections among individual items (or themselves). These paths capture the variance shared between a variable at *t*1 and another (or the same) at *t*2, while all other variables are controlled for. In a first step, we included the estimation of regularised regression coefficients of each variable on itself (autoregressive paths) and on other variables (cross-lagged paths), from *t*1 to *t*2. The LASSO regression used draws on an *l*_1_ penalty via cross-validation, where small regression paths are shrunk to zero, leading to a sparse solution. Nonetheless, LASSO regression biases the non-zero edges towards zero and the cross-validation technique has a high false positive rate to choose the penalty. Thus, in the second-step estimation, the model was re-estimated via a non-regularised regression approach. This was achieved by estimating the model selected in the first-step estimation within an SEM framework using the *lavaan* package^93^. Here, the paths which were estimated to be zero in the regularised regression result (first-step estimation) were held zero. Finally, the model was further pruned by only using the significant parameters from non-regularised model (second-step estimation). We established centrality measures for cross-lagged in-strength, capturing the predictability for each node and is calculated by summing the absolute weights of incoming edges, and for cross-lagged out-strength, capturing the influence for each node, calculated by summing the absolute weights of outgoing edges.

## Supplementary Information

Below is the link to the electronic supplementary material.

Supplementary Material 1

## Data Availability

The data that support the findings of this study are available from the corresponding author, upon reasonable request.

## References

[1] Schweizer, S., Lawson, R. & Blakemore, S. J. Uncertainty as a driver of the youth mental health crisis. *Curr. Opin. Psychol.* **53**, 101657. 10.1016/j.copsyc.2023.101657 (2023). 37517166

[2] Grupe, D. W. & Nitschke, J. B. Uncertainty and anticipation in anxiety: an integrated neurobiological and psychological perspective. *Nat. Rev. Neurosci.* **14**, 488–501 (2013). 10.1038/nrn3524 PMC4276319 23783199

[3] Carleton, R. N. Into the unknown: A review and synthesis of contemporary models involving uncertainty. *J. Anxiety Disord*. **39**, 30–43. 10.1016/j.janxdis.2016.02.007 (2016). 26945765

[4] Sandhu, T. R., Xiao, B. & Lawson, R. P. Transdiagnostic computations of uncertainty: towards a new lens on intolerance of uncertainty. *Neurosci. Biobehav Rev.* **148**, 105123. 10.1016/j.neubiorev.2023.105123 (2023). 36914079

[5] Hedley, F. E., Larsen, E., Mohanty, A., Liu, J. Z. & Jin J.Understanding anxiety through uncertainty quantification. *Br. J. Psychol.* **00**, 1–14. (2024). 10.1111/bjop.12693 PMC13353260 38217080

[6] Insel, T. et al. Research Domain Criteria (RDoC): Toward a New Classification Framework for Research on Mental Disorders. *Am. J. Psychiatry*. **167**, 748–751. 10.1176/appi.ajp.2010.09091379 (2010). 20595427

[7] Afifi, W. A., Felix, E. D. & Afifi, T. D. The impact of uncertainty and communal coping on mental health following natural disasters. *Anxiety Stress Coping*. **25**, 329–347. 10.1080/10615806.2011.603048 (2012). 21801075

[8] Massazza, A., Kienzler, H., Al-Mitwalli, S., Tamimi, N. & Giacaman, R. The association between uncertainty and mental health: a scoping review of the quantitative literature. *J. Ment Health*. **32**, 480–491. 10.1080/09638237.2021.2022620 (2023). 35014927

[9] van der Velden, P. G. et al. Anxiety and depression symptoms, the recovery from symptoms, and loneliness before and after the COVID-19 outbreak among the general population: Findings from a Dutch population-based longitudinal study. *PLoS One*. **16**, e0245057. 10.1371/journal.pone.0245057 (2021). PMC7790276 33411843

[10] World Health Organization. *Mental health and COVID-19: early evidence of the pandemic’s impact: scientific brief, 2 March 2022* (World Health Organization, 2022).

[11] Hertz-Palmor, N. et al. Association among income loss, financial strain and depressive symptoms during COVID-19: Evidence from two longitudinal studies. *J. Affect. Disord*. **291**, 1–8. 10.1016/j.jad.2021.04.054 (2021). PMC8460400 34022550

[12] Monroe, S. M. & Simons, A. D. Diathesis-stress theories in the context of life stress research: implications for the depressive disorders. *Psychol. Bull.* **110**, 406–425. 10.1037/0033-2909.110.3.406 (1991). 1758917

[13] Coyne, J. C. & Downey, G. Social factors and psychopathology: stress, social support, and coping processes. *Annu. Rev. Psychol. ***42**, 401–425 (1991). 10.1146/annurev.ps.42.020191.002153 2018399

[14] Ahir, H., Bloom, N. & Furceri, D. The World Uncertainty Index. *Natl. Bureau Economic Res. Working Paper Ser. *10.3386/w29763 (2022).

[15] Global Burden of Disease Collaborative Network. *Global Burden of Disease Study 2021* (GBD 2021) (Institute for Health Metrics and Evaluation, 2024).

[16] Dugas, M. J., Buhr, K. & Ladouceur, R. The role of intolerance of uncertainty in etiology and maintenance. In *Generalized Anxiety Disorder: Advances in Research And Practice*. (eds Heimberg, R. G.) 143–163 (The Guilford Press, 2004).

[17] Dugas, M. J., Koerner, N. & Freeston, M. H. State of the Science: Intolerance of Uncertainty. *Behav. Ther.* **57**, 17–36. 10.1016/j.beth.2025.08.009 (2026). 41620241

[18] Dugas, M. J., Gagnon, F., Ladouceur, R. & Freeston, M. H. Generalized anxiety disorder: a preliminary test of a conceptual model. *Behav. Res. Ther.* **36**, 215–226. 10.1016/s0005-7967(97)00070-3 (1998). 9613027

[19] McEvoy, P. M., Hyett, M. P., Shihata, S., Price, J. E. & Strachan, L. The impact of methodological and measurement factors on transdiagnostic associations with intolerance of uncertainty: A meta-analysis. *Clin. Psychol. Rev.* **73**, 101778 (2019). 10.1016/j.cpr.2019.101778 31678816

[20] Robichaud, M. Cognitive Behavior Therapy Targeting Intolerance of Uncertainty: Application to a Clinical Case of Generalized Anxiety Disorder. *Cogn. Behav. Pract.* **20**, 251–263. 10.1016/j.cbpra.2012.09.001 (2013).

[21] Dugas, M. J. et al. Behavioral Experiments for Intolerance of Uncertainty: A Randomized Clinical Trial for Adults With Generalized Anxiety Disorder. *Behav. Ther.* **53**, 1147–1160. 10.1016/j.beth.2022.05.003 (2022). 36229113

[22] Morriss, J. Psychological mechanisms underpinning change in intolerance of uncertainty across anxiety-related disorders: New insights for translational research. *Neurosci. Biobehav Rev.* **173**, 106138. 10.1016/j.neubiorev.2025.106138 (2025). 40216169

[23] Wilson, E. J., Abbott, M. J. & Norton, A. R. The impact of psychological treatment on intolerance of uncertainty in generalized anxiety disorder: A systematic review and meta-analysis. *J. Anxiety Disord*. **97**, 102729. 10.1016/j.janxdis.2023.102729 (2023). 37271039

[24] Carleton, R. N., Norton, M. P. J. & Asmundson, G. J. Fearing the unknown: A short version of the Intolerance of Uncertainty Scale. *J. Anxiety Disord*. **21**, 105–117 (2007). 10.1016/j.janxdis.2006.03.014 16647833

[25] American Psychiatric Association DSM-5 Task Force. *Diagnostic and statistical manual of mental disorders: DSM-5™* 5th edn (American Psychiatric Publishing, Inc., 2013).

[26] World Health Organization. International Classification of Diseases, Eleventh Revision (ICD-11). (2019).

[27] Borsboom, D. A network theory of mental disorders. *World Psychiatry*. **16**, 5–13. 10.1002/wps.20375 (2017). PMC5269502 28127906

[28] Newman, M. E. J. Modularity and community structure in networks. *Proc. Natl. Acad. Sci.* **103**, 8577–8582 (2006). 10.1073/pnas.0601602103 PMC1482622 16723398

[29] Andrews, J. L. et al. The effect of intolerance of uncertainty on anxiety and depression, and their symptom networks, during the COVID-19 pandemic. *BMC Psychiatry*. **23**, 261. 10.1186/s12888-023-04734-8 (2023). PMC10109227 37069541

[30] Adamis, A. M., Cole, D. A. & Olatunji, B. O. Intolerance of Uncertainty and Worry Prospectively Predict COVID-19 Anxiety and Distress: A 4-Year Longitudinal Study. *Behav. Ther.* **55**, 320–330. 10.1016/j.beth.2023.07.009 (2024). PMC10902602 38418043

[31] Lei, Y. et al. Intolerance of uncertainty predicts paranoia over time: Evidence from a UK sample. *J. Behav. Ther. Exp. Psychiatry*. **89**, 102050. 10.1016/j.jbtep.2025.102050 (2025). 40782701

[32] Sokol, Y. & Serper, M. Temporal self appraisal and continuous identity: Associations with depression and hopelessness. *J. Affect. Disord*. **208**, 503–511. 10.1016/j.jad.2016.10.033 (2017). 27832930

[33] Sedikides, C., Hong, E. K., Wildschut, T. & Self-Continuity *Annu. Rev. Psychol.* **74**, 333–361 10.1146/annurev-psych-032420-032236 (2023). 35961040

[34] Beck, A. T. *Depression. *(Harper & Row, 1967).

[35] Janoff-Bulman, R. *Shattered assumptions.* (Simon and Schuster, 2010).

[36] Yamagata, M. & Miura, A. Retrospective bias during the COVID-19 pandemic. *Jpn. J. Exp. Soc. Psychol.* **62**, 234–239. 10.2130/jjesp.si5-2 (2023).

[37] Wysocki, A., McCarthy, I., van Bork, R., & Cramer, A.O.J. Cross-lagged panel networks. *advances.in/psychology* **2**, e739621 (2025).

[38] Ramos-Vera, C., García O’Diana, A., Basauri-Delgado, M., Calizaya-Milla, Y. E. & Saintila, J. Network analysis of anxiety and depressive symptoms during the COVID-19 pandemic in older adults in the United Kingdom. *Sci. Rep.* **14**, 7741. 10.1038/s41598-024-58256-8 (2024). PMC10987576 38565592

[39] Schwarz, N. & Sudman, S. *Autobiographical memory and the validity of retrospective reports* (Springer Science & Business Media, 2012).

[40] Buhr, K. & Dugas, M. J. Investigating the construct validity of intolerance of uncertainty and its unique relationship with worry. *J. Anxiety Disord*. **20**, 222–236. 10.1016/j.janxdis.2004.12.004 (2006). 16464706

[41] Koerner, N. & Dugas, M. J. A cognitive model of generalized anxiety disorder: the role of intolerance of uncertainty. In *Worry and its Psychological Disorders: Theory, Assessment and Treatmen*t (eds Davey, G. C. L. & Wells, A.) Ch. 12 (John Wiley & Sons, 2008).

[42] Dugas, M. J., Laugesen, N. & Bukowski, W. M. Intolerance of Uncertainty, Fear of Anxiety, and Adolescent Worry. *J. Abnorm. Child. Psychol.* **40**, 863–870. 10.1007/s10802-012-9611-1 (2012). 22302481

[43] Ren, L. et al. The relations among worry, meta-worry, intolerance of uncertainty and attentional bias for threat in men at high risk for generalized anxiety disorder: a network analysis. *BMC Psychiatry*. **20**, 452. 10.1186/s12888-020-02849-w (2020). PMC7491186 32928164

[44] Mares, L. & Fuller, M. Intolerance of uncertainty and depression in children and adolescents: Systematic review and meta-analysis. *J. Affect. Disord*. **401**, 121288. 10.1016/j.jad.2026.121288 (2026). 41610894

[45] Kroenke, K., Spitzer, R. L., Williams, J. B. W. & Löwe, B. An Ultra-Brief Screening Scale for Anxiety and Depression: The PHQ–4. *Psychosomatics* **50**, 613–621. 10.1016/S0033-3182(09)70864-3 (2009). 19996233

[46] McEvoy, P. M. & Mahoney, A. E. Achieving certainty about the structure of intolerance of uncertainty in a treatment-seeking sample with anxiety and depression. *J. Anxiety Disord*. **25**, 112–122 (2011). 10.1016/j.janxdis.2010.08.010 20828984

[47] Ladouceur, R., Gosselin, P. & Dugas, M. J. Experimental manipulation of intolerance of uncertainty: A study of a theoretical model of worry. *Behav. Res. Ther.* **38**, 933–941 (2000). 10.1016/s0005-7967(99)00133-3 10957827

[48] Shihata, S., McEvoy, P. M., Mullan, B. A. & Carleton, R. N. Intolerance of uncertainty in emotional disorders: What uncertainties remain? *J. Anxiety Disord*. **41**, 115–124 (2016). 10.1016/j.janxdis.2016.05.001 27212227

[49] Rodebaugh, T. L. et al. Does centrality in a cross-sectional network suggest intervention targets for social anxiety disorder? *J. Consult Clin. Psychol.* **86**, 831 (2018). 10.1037/ccp0000336 PMC6166439 30265042

[50] McBride, O. et al. Monitoring the psychological, social, and economic impact of the COVID-19 pandemic in the population: Context, design and conduct of the longitudinal COVID-19 psychological research consortium (C19PRC) study. *Int. J. Methods Psychiatr Res.* **30**, e1861. 10.1002/mpr.1861 (2021). PMC7992290 33166018

[51] Hettich, N. et al. Impact of the COVID-19 pandemic on depression, anxiety, loneliness, and satisfaction in the German general population: a longitudinal analysis. *Soc. Psychiatry Psychiatr. Epidemiol.* **57**, 2481–2490. 10.1007/s00127-022-02311-0 (2022). PMC9181932 35680681

[52] Nitschke, J. B., Heller, W., Imig, J. C., McDonald, R. P. & Miller, G. A. Distinguishing Dimensions of Anxiety and Depression. *Cognit Ther. Res.* **25**, 1–22 (2001).

[53] Borkovec, T. D. The nature, functions, and origins of worry. In *Worrying: Perspectives on Theory, Assessment and Treatment* (eds Davey, G. C. L. & Tallis, F.) 5–33 (John Wiley & Sons, 1994)

[54] Löwe, B. et al. A 4-item measure of depression and anxiety: Validation and standardization of the Patient Health Questionnaire-4 (PHQ-4) in the general population. *J. Affect. Disord*. **122**, 86–95. 10.1016/j.jad.2009.06.019 (2010). 19616305

[55] Kotov, R. et al. The Hierarchical Taxonomy of Psychopathology (HiTOP): A dimensional alternative to traditional nosologies. *J. Abnorm. Psychol.* **126**, 454–477. 10.1037/abn0000258 (2017). 28333488

[56] Hauser, T. U., Skvortsova, V., De Choudhury, M. & Koutsouleris, N. The promise of a model-based psychiatry: building computational models of mental ill health. *Lancet Digit. Health*. **4**, e816–e828 (2022). 10.1016/S2589-7500(22)00152-2 PMC9627546 36229345

[57] Adams, R. A., Huys, Q. J. & Roiser, J. P. Computational psychiatry: towards a mathematically informed understanding of mental illness. *J. Neurol. Neurosurg. Psychiatry*. **87**, 53–63 (2016). 10.1136/jnnp-2015-310737 PMC4717449 26157034

[58] Zhang, Y., Hedley, F. E., Zhang, R. Y. & Jin, J. Toward quantitative cognitive-behavioral modeling of psychopathology: An active inference account of social anxiety disorder. *J. Psychopathol. Clin. Sci.* 10.1037/abn0000972 (2025). 40048236

[59] Buhr, K. & Dugas, M. J. The Intolerance of Uncertainty Scale: psychometric properties of the English version. *Behav. Res. Therarpy*. **40**, 931–945. 10.1016/s0005-7967(01)00092-4 (2002). 12186356

[60] Boswell, J. F., Thompson-Hollands, J., Farchione, T. J. & Barlow, D. H. Intolerance of uncertainty: a common factor in the treatment of emotional disorders. *J. Clin. Psychol.* **69**, 630–645. 10.1002/jclp.21965 (2013). PMC3712497 23381685

[61] Svihra, M. & Katzman, M. A. Behavioural inhibition: A predictor of anxiety. *Paediatr. Child. Health*. **9**, 547–550. 10.1093/pch/9.8.547 (2004). PMC2724161 19680482

[62] Mogg, K., Waters, A. M. & Bradley, B. P. Attention Bias Modification (ABM): Review of Effects of Multisession ABM Training on Anxiety and Threat-Related Attention in High-Anxious Individuals. *Clin. Psychol. Sci.* **5**, 698–717. 10.1177/2167702617696359 (2017). PMC5513441 28752017

[63] Hayes, S. C., Strosahl, K. D. & Wilson, K. G. *Acceptance and commitment therapy: The process and practice of mindful change* 2nd edn (The Guilford Press, 2012).

[64] Beck, A. T. *Cognitive therapy and the emotional disorders* (International Universities Press, 1976).

[65] Huys, Q. J. M., Daw, N. D. & Dayan, P. Depression: A Decision-Theoretic Analysis. *Annu. Rev. Neurosci.* **38**, 1–23. 10.1146/annurev-neuro-071714-033928 (2015). 25705929

[66] Patwardhan, V. et al. Differences across the lifespan between females and males in the top 20 causes of disease burden globally: a systematic analysis of the Global Burden of Disease Study 2021. *Lancet Public Health*. **9**, e282–e294. 10.1016/S2468-2667(24)00053-7 (2024). PMC11080072 38702093

[67] Altemus, M., Sarvaiya, N. & Neill Epperson, C. Sex differences in anxiety and depression clinical perspectives. *Front. Neuroendocrinol.* **35**, 320–330. 10.1016/j.yfrne.2014.05.004 (2014). PMC4890708 24887405

[68] Brewin, C. R., Andrews, B. & Gotlib, I. H. Psychopathology and early experience: a reappraisal of retrospective reports. *Psychol. Bull.* **113**, 82 (1993). 10.1037/0033-2909.113.1.82 8426875

[69] Solhan, M. B., Trull, T. J., Jahng, S. & Wood, P. K. Clinical assessment of affective instability: Comparing EMA indices, questionnaire reports, and retrospective recall. *Psychol. Assess.* **21**, 425–436. 10.1037/a0016869 (2009). PMC2864015 19719353

[70] Morriss, J. et al. Associations between interpersonal trauma and intolerance of uncertainty in an international multi-site sample. *Cognit Ther. Res.* **49**, 185–192. 10.1007/s10608-024-10552-3 (2025).

[71] Sadeh, N. & Bounoua, N. Race moderates the impact of intolerance of uncertainty on mental health symptoms in Black and White community adults. *J. Anxiety Disord*. **93**, 102657. 10.1016/j.janxdis.2022.102657 (2023). PMC9839518 36455413

[72] Pearl, J. *Causality: Models, reasoning, and inference* (Cambridge University Press, 2000).

[73] Pearl, J. The causal mediation formula–a guide to the assessment of pathways and mechanisms. *Prev. Sci.* **13**, 426–436. 10.1007/s11121-011-0270-1 (2012). 22419385

[74] Jacobson, N. C., Chow, S. M. & Newman, M. G. The Differential Time-Varying Effect Model (DTVEM): A tool for diagnosing and modeling time lags in intensive longitudinal data. *Behav. Res. Methods*. **51**, 295–315. 10.3758/s13428-018-1101-0 (2019). PMC6395514 30120682

[75] Gollob, H. F. & Reichardt, C. S. Taking account of time lags in causal models. *Child. Dev.* **58**, 80–92. (1987). 3816351

[76] Epskamp, S. Psychometric network models from time-series and panel data. *Psychometrika* **85**, 206–231 (2020). 10.1007/s11336-020-09697-3 PMC7186258 32162233

[77] Epskamp, S., Borsboom, D. & Fried, E. I. Estimating psychological networks and their accuracy: A tutorial paper. *Behav. Res. Methods*. **50**, 195–212. 10.3758/s13428-017-0862-1 (2018). PMC5809547 28342071

[78] Jach, H. K. & Smillie, L. D. To fear or fly to the unknown: Tolerance for ambiguity and Big Five personality traits. *J. Res. Pers.* **79**, 67–78. 10.1016/j.jrp.2019.02.003 (2019).

[79] Harris, P. A. et al. The REDCap consortium: Building an international community of software platform partners. *J. Biomed. Inf.* **95**, 103208. 10.1016/j.jbi.2019.103208 (2019). PMC7254481 31078660

[80] Talari, K. & Goyal, M. Retrospective studies–utility and caveats. *J. R Coll. Physicians Edinb.* **50**, 398–402 (2020). 10.4997/JRCPE.2020.409 33469615

[81] Moffitt, T. E. et al. How common are common mental disorders? Evidence that lifetime prevalence rates are doubled by prospective versus retrospective ascertainment. *Psychol. Med.* **40**, 899–909. 10.1017/S0033291709991036 (2010). PMC3572710 19719899

[82] Coughlin, S. S. Recall bias in epidemiologic studies. *J. Clin. Epidemiol.* **43**, 87–91. (1990). 10.1016/0895-4356(90)90060-3 2319285

[83] Foygel, R. & Drton, M. Extended Bayesian information criteria for Gaussian graphical models. *Adv Neural Inf. Process. Syst* **23** (2010).

[84] Epskamp, S. & Fried, E. bootnet: Bootstrap methods for various network estimation routines (1.6). *R-CRAN* (2021).

[85] Tibshirani, R. Regression Shrinkage and Selection via the Lasso. *J. Royal Stat. Soc. Ser. B (Methodological)*. **58**, 267–288 (1996).

[86] Epskamp, S., Cramer, A. O. J., Waldorp, L. J., Schmittmann, V. D. & Borsboom, D. qgraph: Network Visualizations of Relationships in Psychometric Data. *J. Stat. Softw.* **48**, 1–18. 10.18637/jss.v048.i04 (2012).

[87] Csárdi, G. et al. igraph: Network analysis and visualization in R (2.1.1). *R-CRAN* (2022).

[88] Newman, M. E. Analysis of weighted networks. *Phys. Rev. E—Statistical Nonlinear Soft Matter Phys.* **70** 10.1103/PhysRevE.70.056131 (2004). 15600716

[89] Jones, P. networktools: Tools for identifying important nodes in networks (1.5.2). *R-CRAN* (2020).

[90] Hallquist, M. N., Wright, A. G. C. & Molenaar, P. C. M. Problems with Centrality Measures in Psychopathology Symptom Networks: Why Network Psychometrics Cannot Escape Psychometric Theory. *Multivar. Behav. Res.* **56**, 199–223. 10.1080/00273171.2019.1640103 (2021). PMC7012663 31401872

[91] van Borkulo, C., Epskamp, S., Jones, P. NetworkComparisonTest: Statistical comparison of two networks based on three invariance measures (2.2.2). *R-CRAN* (2019).

[92] van Borkulo, C. D. et al. Comparing network structures on three aspects: A permutation test. *Psychol. Methods*. 10.1037/met0000476 (2021). 35404628

[93] Rosseel, Y. lavaan: An R package for structural equation modeling. *J. Stat. Softw.* **48**, 1–36 (2012).

