# Supplementary material for: Transdiagnostic symptoms of psychopathology interact with changes in environmental uncertainty
Source: Sci Rep. 2026 Aug 8;16:37327. doi: 10.1038/s41598-026-61219-w (PMC13745279; doi:10.1038/s41598-026-61219-w)
Supplement: Supplementary file 1 [file 41598_2026_61219_MOESM1_ESM.pdf]

	ANX	WOR	DEP	ANH	IUS1	IUS2	IUS3	IUS4	IUS5	IUS6	IUS7	IUS8	IUS9	IUS10	IUS11	IUS12
ANX	1	0.819	0.595	0.446	0.432	0.314	0.302	0.347	0.333	0.342	0.267	0.415	0.411	0.379	0.393	0.362
WOR	***	1	0.571	0.444	0.467	0.341	0.301	0.374	0.325	0.329	0.251	0.43	0.385	0.347	0.381	0.38
DEP	***	***	1	0.709	0.366	0.285	0.206	0.31	0.233	0.291	0.219	0.352	0.449	0.449	0.423	0.401
ANH	***	***	***	1	0.368	0.241	0.154	0.298	0.201	0.28	0.155	0.36	0.379	0.372	0.423	0.4
IUS1	***	***	***	***	1	0.494	0.429	0.612	0.445	0.532	0.359	0.528	0.516	0.517	0.456	0.534
IUS2	***	***	***	***	***	1	0.406	0.397	0.354	0.429	0.365	0.363	0.327	0.415	0.335	0.339
IUS3	***	***	***	**	***	***	1	0.436	0.5	0.459	0.471	0.303	0.265	0.259	0.204	0.268
IUS4	***	***	***	***	***	***	***	1	0.459	0.541	0.382	0.467	0.48	0.481	0.445	0.467
IUS5	***	***	***	***	***	***	***	***	1	0.496	0.529	0.431	0.373	0.385	0.36	0.409
IUS6	***	***	***	***	***	***	***	***	***	1	0.42	0.45	0.377	0.437	0.335	0.487
IUS7	***	***	***	**	***	***	***	***	***	***	1	0.333	0.237	0.331	0.279	0.383
IUS8	***	***	***	***	***	***	***	***	***	***	***	1	0.682	0.619	0.636	0.641
IUS9	***	***	***	***	***	***	***	***	***	***	***	***	1	0.786	0.738	0.632
IUS10	***	***	***	***	***	***	***	***	***	***	***	***	***	1	0.682	0.659
IUS11	***	***	***	***	***	***	***	***	***	***	***	***	***	***	1	0.674
IUS12	***	***	***	***	***	***	***	***	***	***	***	***	***	***	***	1

Supplementary Table S1. Correlation matrix lower environmental uncertainty ($t1$). *Note*: Pearson's correlations of network variables across samples. The correlation coefficient is on the upper diagonal and the strength of the p-value on the lower diagonal. ANX: anxiety/nervousness; WOR: worry; DEP: depression/hopelessness; ANH: anhedonia; IUS1-7: prospective IUS-subscale items; IUS8-12: inhibitory IUS-subscale items; IUS: Intolerance of Uncertainty Scale.
*** $p < .001$, ** $p < .01$.

	ANX	WOR	DEP	ANH	IUS1	IUS2	IUS3	IUS4	IUS5	IUS6	IUS7	IUS8	IUS9	IUS10	IUS11	IUS12
ANX	1	0.768	0.653	0.506	0.439	0.419	0.232	0.341	0.349	0.341	0.184	0.472	0.453	0.45	0.472	0.462
WOR	***	1	0.64	0.557	0.457	0.449	0.257	0.406	0.29	0.362	0.121	0.45	0.437	0.47	0.442	0.473
DEP	***	***	1	0.727	0.401	0.332	0.216	0.363	0.297	0.339	0.15	0.418	0.429	0.444	0.439	0.428
ANH	***	***	***	1	0.361	0.351	0.113	0.371	0.267	0.313	0.103	0.418	0.404	0.444	0.49	0.431
IUS1	***	***	***	***	1	0.547	0.433	0.575	0.489	0.608	0.352	0.554	0.524	0.539	0.462	0.512
IUS2	***	***	***	***	***	1	0.41	0.405	0.427	0.45	0.316	0.418	0.361	0.422	0.358	0.409
IUS3	***	***	***	n.s.	***	***	1	0.446	0.396	0.427	0.476	0.279	0.252	0.288	0.178	0.338
IUS4	***	***	***	***	***	***	***	1	0.447	0.576	0.347	0.452	0.445	0.514	0.455	0.482
IUS5	***	***	***	***	***	***	***	***	1	0.5	0.437	0.48	0.403	0.423	0.342	0.403
IUS6	***	***	***	***	***	***	***	***	***	1	0.42	0.493	0.428	0.489	0.376	0.502
IUS7	**	*	**	n.s.	***	***	***	***	***	***	1	0.279	0.215	0.25	0.261	0.293
IUS8	***	***	***	***	***	***	***	***	***	***	***	1	0.624	0.622	0.612	0.619
IUS9	***	***	***	***	***	***	***	***	***	***	***	***	1	0.773	0.724	0.679
IUS10	***	***	***	***	***	***	***	***	***	***	***	***	***	1	0.714	0.67
IUS11	***	***	***	***	***	***	***	***	***	***	***	***	***	***	1	0.669
IUS12	***	***	***	***	***	***	***	***	***	***	***	***	***	***	***	1

Supplementary Table S2. Correlation matrix higher environmental uncertainty (t2).
Note: Pearson's correlations of network variables across samples. The correlation coefficient is on the upper diagonal and the strength of the *p*-value on the lower diagonal. ANX: anxiety/nervousness; WOR: worry; DEP: depression/hopelessness; ANH: anhedonia; IUS1-7: prospective IUS-subscale items; IUS8-12: inhibitory IUS-subscale items; IUS: Intolerance of Uncertainty Scale.
*** $p < .001$, ** $p < .01$, * $p < .05$, n.s. $p \geq .05$.

	lower environmental uncertainty (*t*1)		higher environmental uncertainty (*t*2)	
item	strength	bridge strength	strength	bridge strength
ANX	0.532	-0.554	0.253	-0.019
WOR	0.649	0.309	1.044	0.758
DEP	0.285	-0.812	0.147	-1.606
ANH	-1.474	-0.129	-0.547	0.655
IUS1	1.185	2.114	1.249	0.884
IUS2	-2.006	-0.506	-1.554	0.096
IUS3	-0.65	-1.429	-0.255	-0.866
IUS4	-0.345	-0.127	-0.507	0.296
IUS5	-0.217	-0.382	-1.032	-0.231
IUS6	-0.097	-0.186	0.356	-0.47
IUS7	-1.423	-1.133	-2.171	-1.707
IUS8	-0.059	1.284	-0.182	2.006
IUS9	1.532	-0.563	0.66	-1.302
IUS10	1.003	0.737	1.384	0.463
IUS11	0.392	-0.366	0.946	0.276
IUS12	0.693	1.742	0.209	0.769

Supplementary Table S3. Centralities for both networks.
Note: Standardised centrality indices of strength (i.e., sum of the absolute weights of every edge) and bridge strength (i.e., sum of absolute weights of edges across clusters) of cross-sectional networks at both timepoints (*t*1 and *t*2). ANX: anxiety/nervousness; WOR: worry; DEP: depression/hopelessness; ANH: anhedonia; IUS1-7: prospective IUS-subscale items; IUS8-12: inhibitory IUS-subscale items; IUS: Intolerance of Uncertainty Scale.

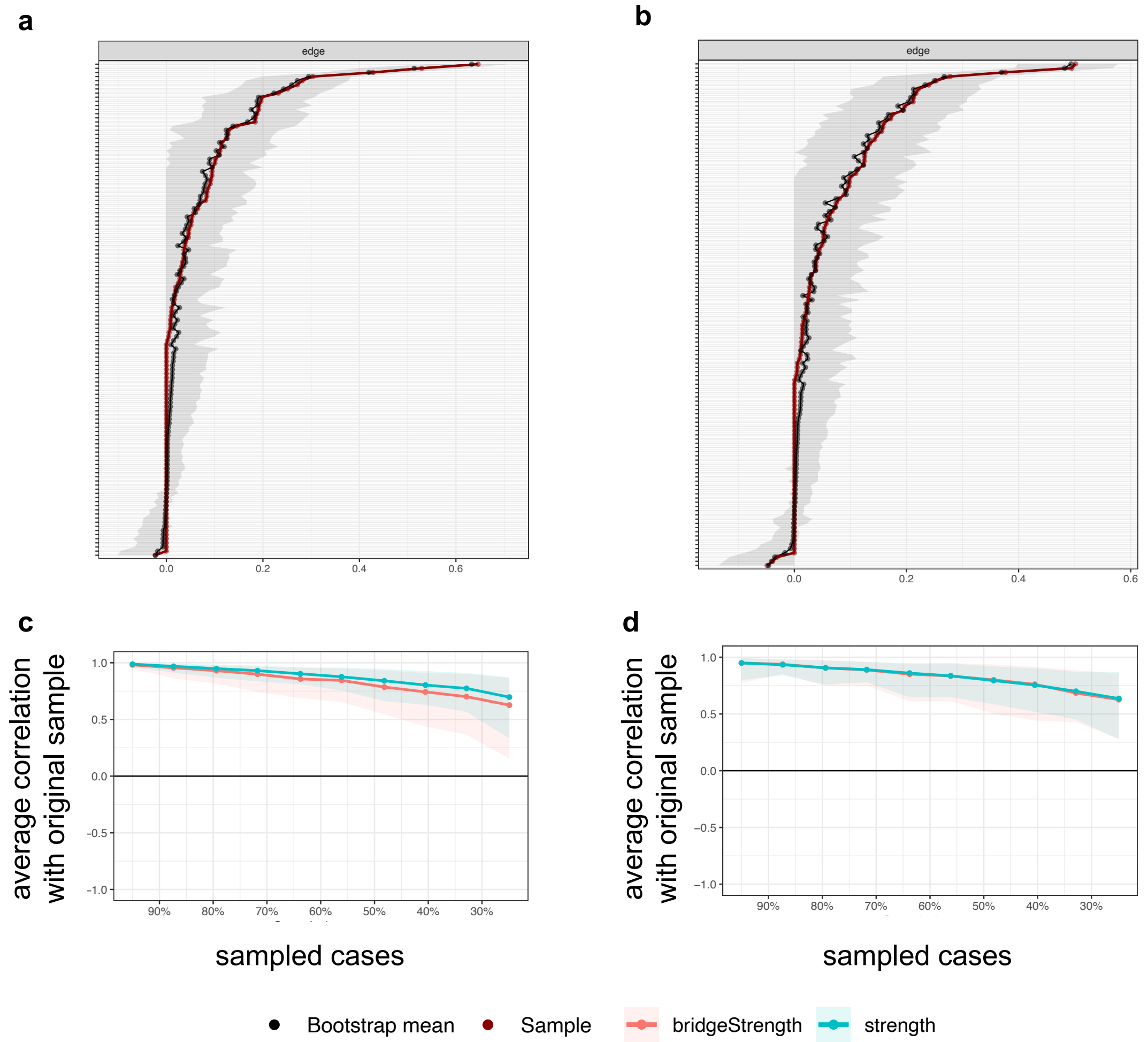

Supplementary Fig S1. Bootstrapped stability for cross-sectional networks.
Note: (**a**) and (**b**) Accuracy of the edges weights established via non-parametric bootstrapped resampling for the two cross-sectional networks for (**a)** lower environmental uncertainty (*t*1) and (**b**) higher environmental uncertainty (*t*2). Red dots and lines: edge weights from the samples. Black dots and lines: edge weights generated based on 2000 random bootstrap samples. Gray shades: bootstrap-generated confidence intervals for each edge weight. **c** and (**d**) Correlation stability of centrality indices established via case-dropping bootstrapped resampling for (**c**) lower environmental uncertainty (*t*1) and (**d**) higher environmental uncertainty (*t*2). Solid lines link the means of correlations from subsets with increasing number of excluded cases. Colored areas indicate the range of correlations between 2.5th quantile and 97.5th quantile.

lower environmental uncertainty (*t*1) \ higher environmental uncertainty (*t*2)	ANX	WOR	DEP	ANH	IUS1	IUS2	IUS3	IUS4	IUS5	IUS6	IUS7	IUS8	IUS9	IUS10	IUS11	IUS12
ANX	0.289	0	0	0	0	0	0	0	0	-0.058	0	0	0	0	0	0
WOR	0	0.343	0	0	0	0	0	0	0	0	0	0	0	0	0	0
DEP	0	0	0.413	0	0	-0.179	0	0	0	0	0	0	-0.08	0	-0.066	-0.083
ANH	0	0.082	0	0.296	0	0.164	0	0	0	0.086	0	0	0	0	0	0.062
IUS1	0	0	0	0	0.699	0	0	0	0	0	0	0	0	0	0	0
IUS2	0	0	0	0	0	0.724	0	0	0	0.051	0	0	0	0	0	0
IUS3	0	0	0	0	0	0	0.763	0	-0.117	-0.066	0	-0.142	-0.101	0	-0.069	0
IUS4	0	0	0	0	-0.081	-0.096	0	0.62	0	0	0	0	0	0	0	-0.062
IUS5	0	0	0	0	0	0.078	0	0	0.837	0	0	0	0	0	0	0
IUS6	0	0	0	0	0.073	0	0	0	0	0.821	0	0	0	0	0	0
IUS7	0	0	0	0	0	0	0	0	0	0	0.566	0.11	0.068	0	0.079	0.063
IUS8	0	0	0	0	0	0	0	0	0	0.061	0	0.643	0	0	0	0
IUS9	0	0	0	0	0	0	0	0	0	0	0	-0.13	0.746	0	0	0
IUS10	0	0	0	0	0.117	0	0	0	0	0	0	0	0.114	0.794	0.125	0
IUS11	0.221	0	0	0.171	0	0	0	0	0	0	0	0	0	0.096	0.782	0.072
IUS12	0	0	0	0	0	0	0.096	0.108	0	0	0	0	0	0	0	0.813

Supplementary Table S4. Standardised regression matrix of CPLN model.
Note: Edge weight results of the cross-lagged panel network from lower environmental uncertainty (rows) to higher environmental uncertainty (column). Diagonal items are autoregressive values. ANX: anxiety/nervousness; WOR: worry; DEP: depression/hopelessness; ANH: anhedonia; IUS1-7: prospective IUS-subscale items; IUS8-12: inhibitory IUS-subscale items; IUS: Intolerance of Uncertainty Scale.

item	in-strength	out-strength
ANX	0.221	0.058
WOR	0.082	0
DEP	0	0.409
ANH	0.171	0.395
IUS1	0.271	0
IUS2	0.517	0.051
IUS3	0.096	0.495
IUS4	0.108	0.239
IUS5	0.117	0.078
IUS6	0.322	0.073
IUS7	0	0.32
IUS8	0.382	0.061
IUS9	0.364	0.13
IUS10	0.096	0.356
IUS11	0.339	0.559
IUS12	0.343	0.204

Supplementary Table S5. In-strength and out-strength in the CLPN network. *Note*: Indices of in-strength (predictability, i.e., sum of absolute weights of incoming edges) and out-strength (influence, i.e., sum of absolute weights of outgoing edges) of cross-lagged panel network (CLPN). ANX: anxiety/nervousness; WOR: worry; DEP: depression/hopelessness; ANH: anhedonia; IUS1-7: prospective IUS-subscale items; IUS8-12: inhibitory IUS-subscale items; IUS: Intolerance of Uncertainty Scale.

PHQ-4

**Model**	**χ² (df)**	**CFI**	**RMSEA (90% CI)**	**SRMR**	**ΔCFI**	**ΔRMSEA**
Configural	209.77 (15)	0.876	0.207 [0.181–0.234]	0.079	—	—
Metric	236.33 (18)	0.861	0.200 [0.176–0.224]	0.090	−0.015	−0.007

IUS-12

**Model**	**χ² (df)**	**CFI**	**RMSEA (90% CI)**	**SRMR**	**ΔCFI**	**ΔRMSEA**
Configural	491.42 (234)	0.971	0.054 [0.044–0.063]	0.067	—	—
Metric	503.54 (244)	0.971	0.053 [0.044–0.062]	0.068	0.000	−0.001

Supplementary Table S6. Measurement invariance results for PHQ-4 and IUS-12 across timepoints.
Note: ΔCFI and ΔRMSEA represent changes relative to the configural model. Values within |ΔCFI| ≤ .01 and |ΔRMSEA| ≤ .015 are typically considered evidence of metric invariance.

Supplementary Table S6 reports full measurement invariance results for the PHQ-4 and IUS-12 across timepoints. Configural invariance was supported for both measures, indicating that the same factor structures were identifiable across time. For the IUS-12, metric invariance was also fully supported, with small changes in model fit relative to the configural model. For the PHQ-4, metric invariance showed only minor changes in approximate fit indices (ΔCFI = −0.015; ΔRMSEA = −0.007), suggesting broadly comparable factor loadings across timepoints despite some model sensitivity. Overall, results indicate strong longitudinal measurement stability for the IUS-12 and acceptable stability for the PHQ-4, supporting the use of both instruments in longitudinal analyses of symptom associations.
